# Comprehensive clinicopathological and genomic profiling of gallbladder cancer reveals actionable targets in half of patients

**DOI:** 10.1038/s41698-022-00327-y

**Published:** 2022-11-05

**Authors:** Tessa J. J. de Bitter, Philip R. de Reuver, Elise A. J. de Savornin Lohman, Leonie I. Kroeze, Marianne E. Vink-Börger, Shannon van Vliet, Femke Simmer, Daniel von Rhein, Erik A. M. Jansen, Joanne Verheij, Carla M. L. van Herpen, Iris D. Nagtegaal, Marjolijn J. L. Ligtenberg, Rachel S. van der Post

**Affiliations:** 1 Radboud University Medical Center, Radboud Institute for Molecular Life Sciences, Department of Pathology, Nijmegen, The Netherlands; 2 Radboud University Medical Center, Radboud Institute for Health Sciences, Department of Surgery, Nijmegen, The Netherlands; 3 Radboud University Medical Center, Radboud Institute for Molecular Life Sciences, Department of Human Genetics, Nijmegen, The Netherlands; 4 Cancer Center Amsterdam, Amsterdam UMC, University of Amsterdam, Department of Pathology, Amsterdam, The Netherlands; 5 Radboud University Medical Center, Radboud Institute for Health Sciences, Department of Medical Oncology, Nijmegen, The Netherlands

**Keywords:** Cancer genomics, Tumour biomarkers

## Abstract

Gallbladder cancer (GBC) is a rare, highly aggressive malignancy with a 5-year survival rate of 5–10% in advanced cases, highlighting the need for more effective therapies. The aim of this study was to identify potentially actionable therapeutic targets for GBC. Specimens and clinicopathological data of 642 GBC patients, diagnosed between 2000 and 2019 were collected using the Dutch Pathology Registry (PALGA) and the Netherlands Cancer Registry. All cases were histologically reviewed and a subset was subjected to a comprehensive next generation sequencing panel. We assessed mutations and gene amplifications in a panel of 54 actionable genes, tumor-mutational burden (TMB), and microsatellite instability (MSI). Additionally, the entire cohort was screened for HER2, PD-L1, pan-TRK, and p53 expression with immunohistochemistry. Histopathological subtypes comprised biliary-type adenocarcinoma (AC, 69.6%), intestinal-type AC (20.1%) and other subtypes (10.3%). The median total TMB was 5.5 mutations/Mb (range: 0–161.1) and 17.7% of evaluable cases had a TMB of >10 mutations/Mb. MSI was observed in two cases. Apart from mutations in *TP53* (64%), tumors were molecularly highly heterogeneous. Half of the tumors (50%) carried at least one molecular alteration that is targetable in other tumor types, including alterations in *CDKN2A* (6.0% biallelically inactivated), *ERBB2* (9.3%) and *PIK3CA* (10%). Immunohistochemistry results correlated well with NGS results for HER2 and p53: Pearson *r* = 0.82 and 0.83, respectively. As half of GBC patients carry at least one potentially actionable molecular alteration, molecular testing may open the way to explore targeted therapy options for GBC patients.

## Introduction

Gallbladder cancer (GBC) is an aggressive cancer and is often diagnosed at an advanced stage, with a 5-year survival rate of 5–10%^1–3^. Complete surgical resection is the only treatment with curative intent, for which only about 10–20% of patients are eligible at time of diagnosis^4^.

Evidently, there is a dire need for innovative systemic treatment options for GBC. Current molecular profiling studies either originate from endemic regions with presumed different etiology or constitute small case series with the use of relatively limited targeted next generation sequencing (NGS) panels. High-throughput sequencing studies are critical to evaluate the potential of genome-informed treatment selection in GBC.

During the last decade, molecular medicine has increasingly been incorporated in disease management of various cancer types. Besides two pan-cancer markers, the FDA recognized 37 genetic biomarkers for the prediction of response to an FDA-approved drug in 19 different solid tumor types (OncoKB Level 1), and another 12 standard care biomarkers are recommended by professional guidelines as predictive of response to an FDA-approved drug in 11 different solid tumor types (OncoKB Level 2). (OncoKB, accessed 03-07-2021^5^). For GBC there are currently no specific predictive biomarkers recognized, except for biomarkers that apply to all solid tumors; *NTRK1-3* fusions, microsatellite instability (MSI)-high or tumor mutational burden (TMB)-high. (OncoKB, accessed 03-07-2021^5^).

To expand putative treatment options for patients with GBC, the aim of this study was to identify actionable molecular targets for GBC, which is a rare disease in Western countries with limited molecular data. Therefore, we performed an integrative clinical, histopathological, and molecular analysis in a nationwide cohort of 642 GBC patients. A subset of tumors was subjected to a comprehensive NGS panel that simultaneously detects variants in 523 cancer-related genes on DNA level, and fusion genes and splice variants of 55 genes on RNA level. In addition, presence of a subset of potential therapeutic targets was assessed on the protein level by immunohistochemistry (IHC).

## Results

### Patient and tumor characteristics

**Fig. 1:**
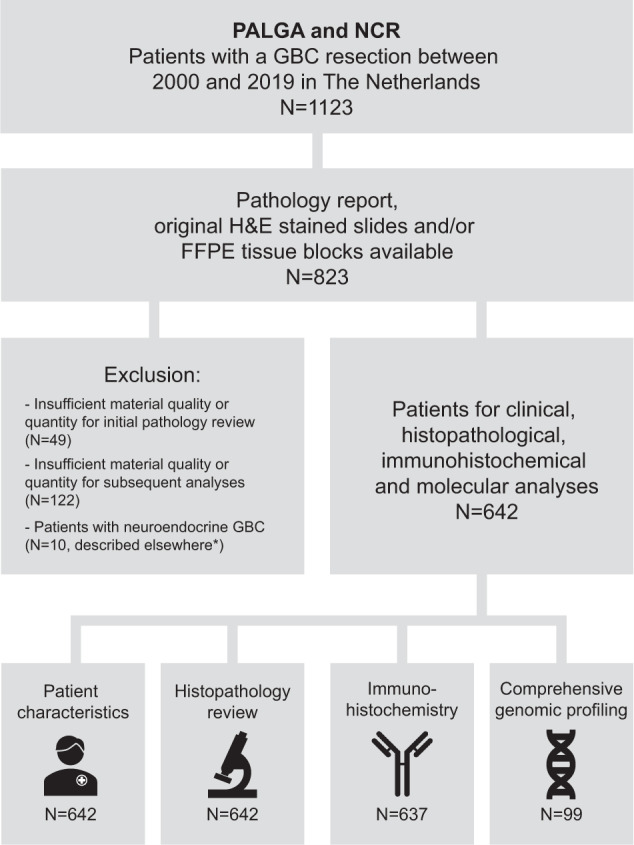
Flowchart of the study. Patients were anonymously selected using PALGA and the NCR and their clinical, histopathological and molecular characteristics were analysed. PALGA Dutch Nationwide Pathology Databank, NCR Netherlands Cancer Registry, H&E hematoxylin and eosin, FFPE Formalin-Fixed Paraffin-Embedded. *Patients with neuro-endocrine GBC were described previously^21^.

Between 2000 and 2019, 1123 patients were identified who underwent a resection for primary GBC in the Netherlands, of which 642 patients were eligible for subsequent analyses (Fig. 1).

**Fig. 2:**
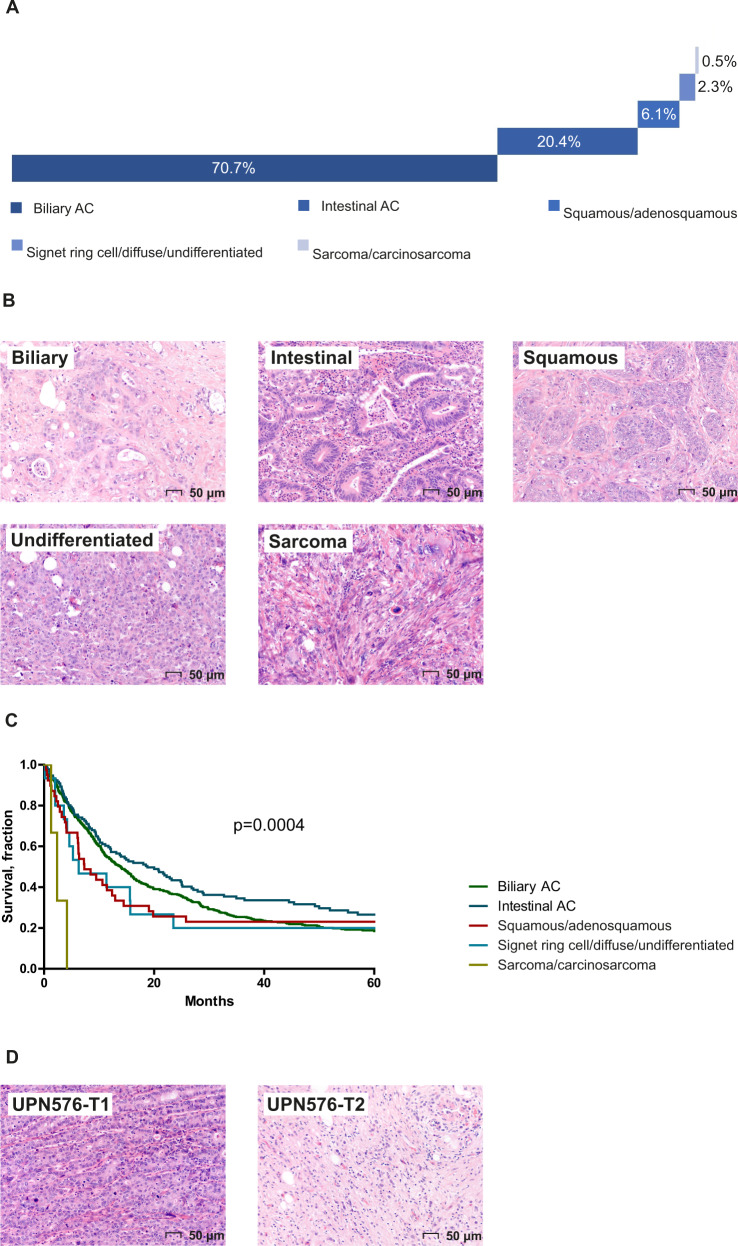
Histological subtypes of GBC (*N* = 642). **A** Distribution of histological subtypes; **B** representative hematoxylin and eosin stained slides of each subtype; **C** survival according to histological subtype; **D** two distinct morphological regions within the same tumor, with UPN576-T1 showing a predominantly solid undifferentiated growth pattern and UPN576-T2 showing a predominantly diffuse growth pattern.

The median age at diagnosis was 69 years (SD ± 11 years) and 73% of patients were female (Table 1). The majority of tumors were diagnosed at pathological stage 2 (pT2, 60.8%) followed by stage pT3 (28.7%), pT1 (8.9%) and pT4 (0.8%). For six patients (0.9%) T-stage could not be assessed (pTx), because the resection specimen was incomplete or fragmented. Lymph node involvement could not be assessed in the majority of cases (70.6%), because this information in the original pathology report was lacking or no lymph node was resected or identified macroscopically^6^. The majority of tumors were biliary-type adenocarcinoma (AC, 70.7%), followed by intestinal-type AC (20.4%) and other less common subtypes. About half of the cases were high grade (44.5%) and a substantial proportion of cases showed venous-, lymphatic-, or perineural invasion (Table 1, Fig. 2, and Supplementary Data 1).

**Table 1:** Patient and tumor characteristics.

	Total cohort (*n* = 642)	NGS cohort (*n* = 99)^a^	*P*-value
Age (mean, SD)	69 ± 11.5	67 ± 12.0	
Gender (female)	470 (73.2%)	69 (69.7%)	n.s.
pT classification			
T1a	9 (1.4%)	0 (0.0%)	n.s.
T1b	48 (7.5%)	0 (0.0%)	0.0049
T2nos	145 (22.6%)	14 (14.1%)	n.s.
T2a	123 (19.2%)	18 (18.2%)	n.s.
T2b	122 (19.0%)	26 (26.3%)	n.s.
T3	184 (28.7%)	38 (38.4%)	0.0493
T4	5 (0.8%)	2 (2.0%)	n.s.
Tx	6 (0.9%)	1 (1%)	n.s.
pN classification			
N0	77 (12.0%)	18 (18.2%)	n.s.
N1	112 (17.4%)	30 (30.3%)	0.0025
Nx	453 (70.6%)	51 (51.5%)	0.0002
Histology			
Biliary AC	454 (70.7%)	71 (71.7%)	n.s.
Intestinal AC	131 (20.4%)	17 (17.2%)	n.s.
Squamous/adenosquamous carcinoma	39 (6.1%)	7 (7.1%)	n.s.
Signet ring cell/diffuse/undifferentiated AC	15 (2.3%)	4 (4.0%)	n.s.
Carcinosarcoma/sarcoma	3 (0.5%)	0 (0.0%)	n.s.
Tumor grade			
Low	356 (55.5%)	50 (50.5%)	n.s.
High	286 (44.5%)	49 (49.5%)	n.s.
Venous invasion (yes)	219 (34.1%)	56 (56.6%)	<0.0001
Lymphatic invasion (yes)	294 (45.8%)	70 (70.7%)	<0.0001
Perineural invasion (yes)	212 (33.0%)	54 (54.5%)	<0.0001

Differences between categorical variables in the NGS cohort and the total cohort (including NGS cohort) were assessed by two-tailed Χ^2^ test and *P*-values of <0.05 were considered statistically significant.

^a^*n* = 100 samples were included in the NGS cohort, corresponding with 99 patients.

The median overall survival (OS) was 13.6 months (95% CI: 11.7–15.5) across all histological subtypes; survival was best in patients with an intestinal-type AC (median OS 18.8 months, 95% CI: 12.0–25.6), whereas the poorest OS was observed in patients with a sarcoma/carcinosarcoma-type GBC (median OS 2.4 months, 95% CI: 0.6–4.1) (Fig. 2C), log-rank *p* = 0.0004.

### Molecular analysis

DNA and RNA were isolated from 100 tumor samples (99 patients) and 98 tumor samples (97 patients), respectively; for two samples material was insufficient for RNA extraction. The NGS cohort was considered a representative subgroup of the total cohort as there were no statistically significant differences observed for the majority of clinicopathological characteristics (Table 1). The tumor of one patient showed two distinct histological regions within the same tumor, which were isolated and sequenced separately (Fig. 2D). For four DNA samples the TMB value and MSI status could not be determined, and for two additional samples MSI status could not be determined due to insufficient coverage. Nine RNA samples did not meet quality control criteria (Supplementary Data 2).

**Fig. 3:**
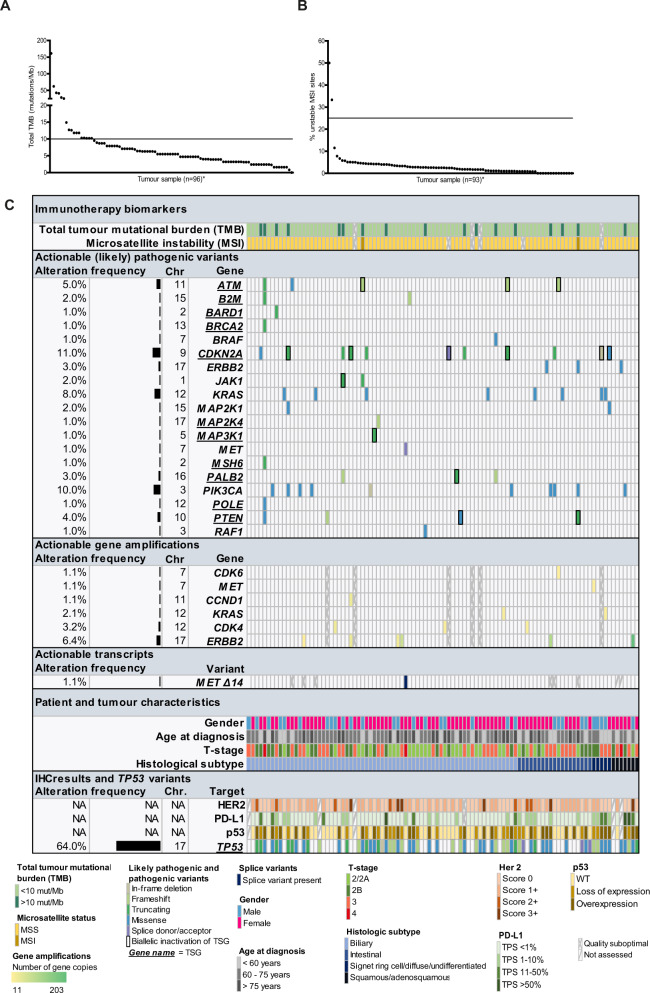
Potential therapeutic targets in GBC. **A** Total tumor mutational burden (TMB), **B** Microsatellite instability (MSI), **C** Integrative overview of immunotherapy biomarkers (TMB, MSI), clinically relevant mutations (class 4 and 5), gene amplifications, transcripts and immunohistochemistry results. NA not applicable.

To predict response to immunotherapy, the TMB and MSI status of each tumor was evaluated. The median total TMB was 5.5 mutations/Mb (range: 0–161.1 mutations/Mb) and in 17.7% (17 out of 96 evaluable cases) the total TMB was ≥ 10 mutations/Mb (“TMB-high”) (Fig. 3A, C). The tumor with the highest TMB (161.1 mutations/Mb) had a pathogenic *POLE* variant. Two tumors were MSI-High with >25% unstable MSI sites. These were among the four tumors with the highest TMB (Fig. 3B, C; Supplementary Data 2). In neither MSI-high tumor, a pathogenic variant in a DNA mismatch repair (MMR) gene was detected. Immunohistochemical analysis of the MMR proteins at our diagnostics laboratory showed absence of MLH1 and PMS2 staining in both tumors. In one tumor the MLH1 promoter showed hypermethylation; in the other tumor the MLH1 promotor was unaffected. As therapeutic targeting of TSG typically requires inactivation of both alleles, for each inactivating mutation in a TSG, it was checked whether it likely concerned monoallelic or biallelic inactivation (Fig. 3C and Supplementary Data 3). Likely pathogenic or pathogenic single- and multiple nucleotide variants (classes 4 and 5) were observed in 33.0% of tumors in a variety of actionable target genes (Fig. 3C). The most frequently altered gene was *TP53* (64%, not considered actionable), followed by actionable target genes *CDKN2A* (6%, biallelically inactivated), *PIK3CA* (10%) and *KRAS* (8%), including two *KRAS* p.G12C variants which are now considered targetable in other tumor types. A variety of other genes carried class 4 and 5 variants mainly in single cases.

Potentially actionable gene amplifications were observed in 13.8% of tumors in a variety of genes including *CDK4*, *CDK6*, *CCND1*, *ERBB2, KRAS*, and *MET* (Fig. 3C). Interestingly, a *MET* amplification was observed in the tumor with two morphologically distinct components: the histological component with a predominantly solid undifferentiated-type growth pattern carried the amplification whilst the other with a predominantly diffuse growth pattern did not (Fig. 2C). Both histological components shared a pathogenic variant in *TP53*.

A potentially actionable splice donor site variant in intron 14 of *MET* (ENST00000318493.10:c.3082+1>C) was confirmed to lead to skipping of exon 14 on RNA level. Apart from this *MET* splice variant, no other splice variants or fusion transcripts were detected in our cohort in a panel of 55 genes, including amongst others *ALK*, *ROS1*, and *RET* (Supplementary Data 3).

Collectively, half of GBC patients (50%) carried at least one molecular alteration that is targetable in other tumor types, including TMB-High, MSI-High, gene amplification, and (likely) pathogenic variant (with biallelic inactivation in case of TSGs). No statistically significant differences were observed among histological subtypes in terms of frequency of actionable molecular therapeutic targets. When comparing biliary vs. intestinal vs. ‘other’ type (i.e., squamous/adenosquamous carcinoma and signet ring cell/diffuse/undifferentiated carcinoma), significantly more class 4/5 *TP53* alterations were observed in the biliary type adenocarcinoma cases (*P* = 0.000).

### Immunohistochemical analysis of therapy targets

**Fig. 4:**
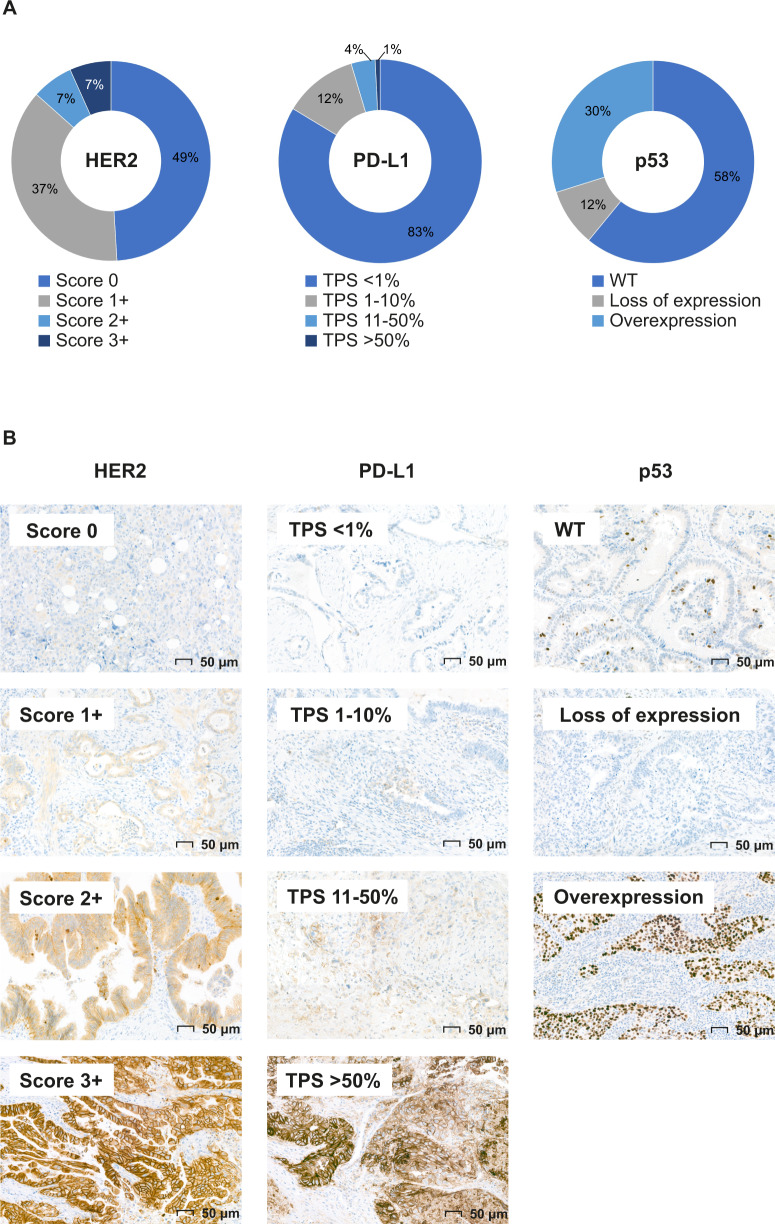
Potential therapeutic targets on the protein level in GBC (*N* = 637). **A** Distribution of immunohistochemistry (IHC) results. **B** Representative images of IHC scoring. TPS tumor proportion score.

Immunohistochemical results were available for 637 out of 642 patients, including 95 NGS cases. Depending on the stain, up to 20 TMA cores were not assessable due to sampling error (only tumor stroma) or absence of the core (Supplementary Data 1). A HER2 IHC score of 3+ (overexpression) was observed in 6.6% of tumors (Fig. 4). PD-L1 positivity, expressed as tumor proportion score (TPS) 1–10%, TPS 11–50% and TPS > 50% was observed in 12.0% and 3.9% and 0.8% of tumors, respectively (Fig. 4). Aberrant p53 expression was observed in 42.1% of tumors; either complete loss (11.6%) or overexpression (30.5%) (Fig. 4). One case (0.2%) showed pan-TRK immuno-expression, which was not confirmed by fusion transcript detection on RNA level.

Altogether, in 23.3% of tumors a potential therapeutic target (i.e., HER2 score 3+ or PD-L1 TPS ≥ 1%) was identified on the protein level.

### Integrative analysis of NGS and IHC findings

For 95 patients that underwent NGS and IHC, findings were compared. Overall, IHC data correlated well with NGS data for HER2 (*ERBB2*) and p53 (*TP53*): Pearson *r* = 0.82 and 0.83, respectively. One case with heterogeneous regions with HER2 overexpression (IHC score 3+) was confirmed with fluorescence in situ hybridization, but an *ERBB2* gene amplification could not be detected by NGS. In addition, one case with a low-level *ERBB2* gene amplification on NGS level (estimated gene copies: 11) could not be confirmed on the protein level. In eight out of 65 mutant *TP53* cases (12.3%), a wild-type IHC pattern was observed. The variant allele frequency (VAF) of these variants ranged from 11 to 25% and included truncating variants, missense variants, in-frame deletions, and splice site variants (*N* = 2 for each variant type). In addition, one case with p53 overexpression on IHC carried a missense variant of unknown significance in *TP53*. In eight out of 16 cases (50%) with “TMB-High” for which IHC data was also available, the PD-L1 TPS score was >1%; the other cases with “TMB-high” had a PD-L1 TPS score <1%. The single case with pan-TRK immune-expression was subjected to the Archer FusionPlex lung panel assay at our diagnostics laboratory, but results were not sufficient to reliably exclude the presence of an *NTRK* fusion gene.

## Discussion

We investigated the clinicopathological and molecular characteristics of GBC in a low incidence population. Whereas GBC is traditionally considered a tumor with an extremely poor prognosis and with limited treatment options^7^, this study identified at least one potentially actionable therapeutic target in 50.0 and 23.3% of tumors on the DNA and protein level, respectively. This may provide an essential basis for future personalized therapy in GBC patients.

The emergence of immunotherapy is one of the major breakthroughs in cancer treatment of the past decade. Several studies have demonstrated the predictive value of TMB and MSI as biomarkers for the efficacy of treatment with immune checkpoint inhibitors^8,9^. The optimal threshold for TMB-High is likely cancer type dependent^10^ and still has to be established in clinical trials for many cancer types, including GBC. For lung cancer, this threshold was set at 10 mutations/Mb in the CheckMate 568 trial^11^. Although the median total TMB in the present study was relatively low (5.5 mutations/Mb), in 17.7% of cases the TMB was ≥10 mutations/Mb (“TMB-High”). Of note, the only two tumors that were MSI-High in this cohort were among the four tumors with the highest total TMB. This is also seen in other cancer types with MSI-high tumors^12^. A third approved predictive biomarker for immunotherapy is PD-L1 expression^13^, which was observed with varying frequency in 16.7% of cases. Although in 56% of cases with TMB-high the PD-L1 TPS-score was >1%, there was no clear correlation between PD-L1 status and TMB. The optimal PD-L1 scoring method is matter of debate, since no trial data on the effect of immunotherapy and PD-L1 expression in GBC are available. Therefore the optimal cut-off is unknown regarding expression score, including TPS versus combined positive score (CPS) which includes inflammatory cells as well as the preferred antibody clone.

In a large variety of ‘actionable’ genes, (likely) pathogenic variants were observed in 39% of patients, and 13% of cases carried at least one gene amplification for which therapies are available in other tumor types. For example, alterations of *ERBB2* (amplifications or oncogenic variants) were observed in 9.4% of patients and are targetable in multiple cancers with a variety of anti-HER2 (combination) therapies^5^. HER2 IHC results correlated well with NGS results (*r* = 0.83), revealing HER2 overexpression in 6.7% of cases, and can therefore serve as surrogate marker to detect *ERBB2* gene amplifications in GBC. Alterations of the *ERBB2* pathway have been observed before in other ‘endemic’ GBC populations with similar frequencies^14,15^. Currently, six phase 2 clinical trials are ongoing, that include amongst others GBC patients, targeting (members of) the *ERBB2* signaling pathway (clinicaltrials.gov, accessed 03-10-2021: NCT03768375, NCT04183712, NCT02091141, NCT04430738, NCT04579380, NCT04466891).

A single *METΔ14* splice variant was observed, resulting in skipping of exon 14 of *MET*. This leads to decreased MET degradation and increased downstream signaling^16^. This variant has been identified as oncogenic driver in non-small cell lung cancer patients and is targetable with for example capmatinib, tepotinib, or crizotinib^5,17^. Interestingly, no gene fusions were observed in this cohort in a panel of 55 tested genes. In addition, no NTRK fusion genes were detected on the protein level in the entire cohort, reflected by negative pan-TRK IHC in all but one case which could not be confirmed with NGS. This is in sharp contrast to what is known for intrahepatic cholangiocarcinomas, that have *FGFR2* fusions in 10–16% of cases that may be sensitive to infigratinib and pemigatinib^5,18–20^. We previously reported an *FGFR3-TACC3* fusion in a case with neuroendocrine GBC^21^, but outside this example fusion genes appear not to play a major role in GBC.

Our data underscore the intertumoral heterogeneity that has been observed in previous studies of biliary tract cancers^22–24^, complicating the study of efficacy of treatments stratified on these molecular alterations. Despite this heterogeneity, some actionable targets seem more prevalent, such as *ERBB2* alterations, and their prevalence appears similar in both high- and low incidence populations^23^. Intratumoral heterogeneity, reflected by the presence of a *MET* amplification in only one morphological region of the tumor but not the other, poses an additional challenge in assessing whether a given genetic alteration reflects an essential pathway that can serve as a molecular target. In order to appropriately assess this intratumoral heterogeneity, extensive sampling for accurate histopathological assessment is essential to provide a complete overview of the resection specimen and select representative areas for nucleic acid extraction for molecular analyses.

A major strength of this study is the use of a nation-wide cohort of a low-incidence population of GBC patients, which is highly limited in literature. Based on a systematic literature search^25^, only nine studies with *N* ≥ 100 patients were published^14,15,26–32^, and five were derived from endemic regions (Chile, China, India), with different etiology^14,26,27,30,31^. The other four were derived from the United States, but included patients from various ethnicities^15^, unspecified ethnicity^28^, or included primary and metastatic GBC lesions^29,32^, which render them not directly comparable to our study. Overall, we believe this study demonstrates novelty, since this study includes clinicopathological and immunohistochemical analyses from over 600 patients and molecular data from 100 tumor samples. A large, systematically analyzed Western cohort has not been published before. Another strength is the use of a comprehensive DNA- and RNA-based NGS panel that simultaneously assessed the most important cancer biomarkers in a single assay, which is validated and implemented in routine diagnostics in our laboratory^33^, and the integration with IHC results. Results of this study significantly contribute to the molecular understanding of GBC in low incidence populations and can expand current treatment possibilities by allowing GBC patients to enter basket trials based on their molecular profile such as the The Drug Rediscovery Protocol (DRUP Trial), initiated in the Netherlands (Clinicaltrials.gov, NCT02925234).

Due to the retrospective, anonymized nature of the study, patients were not able to receive genome-informed treatment, which is a limitation of this study. Another limitation is that the distinction between mono-and biallelic inactivation of TSGs was difficult in a number of cases, which might have led to an underestimation of potentially targetable variants.

In conclusion, this study demonstrates that although GBC appears molecularly heterogeneous, half of the tumors harbor potentially actionable alterations on the genomic level and 23% on the protein level for selected targets. Broad molecular testing may lead to improved treatment options for a significant number of GBC patients.

## Methods

### Patient selection

Patients that had undergone a resection for GBC between 2000 and 2019 were anonymously selected using the linkage between the nationwide network and registry of histopathology and cytopathology in the Netherlands (PALGA, LZV2017-87)^34^ and the Netherlands Cancer Registry (NCR, K171236).

Baseline information on gender, age at time of diagnosis, and vital status including follow-up time was provided by the NCR and supplemented with information from the original pathology reports. This study was approved by the local Radboud university medical center medical ethics committee (2018-1426). A waiver of consent for this specific study was granted because data of patients who, during their treatment, object to the use of their data for scientific research are not included in PALGA.

### Patient selection for NGS and nucleic acid extraction

Sample selection for NGS was based on: 1: estimated percentage of neoplastic cells of ≥30% to ensure accurate MSI and TMB assessment^33^, 2: pathological T-stage ≥pT2a (TNM 8th edition; invasion of the perimuscular connective tissue), 3: year of diagnosis sorted on most recent first and limited to 100 samples.

DNA and RNA were extracted from formalin-fixed paraffin-embedded tissue and precipitated using 5% Chelex-100 in TET-lysis buffer and proteinase K for DNA isolation, and the ReliapPrep FFPE total RNA Miniprep System for RNA isolation (Promega, Madison, WI), as described previously^21^. Final concentrations were determined by Qubit DNA/RNA high sensitivity kits (Thermo Fisher Scientific, Waltham, MA) according to the manufacturer’s instructions. Subsequently, either 60–100 ng of DNA or 60 ng of RNA was used as input for the NGS library preparation.

### Library preparation, sequencing, and analysis

Targeted NGS libraries were prepared using the Trusight Oncology 500 (TSO500) DNA and RNA library preparation kits (Illumina, San Diego, CA). Library preparation, sequencing, analysis of gene amplifications, variant annotation, and variant filtering were extensively described previously^21^. Briefly, DNA samples were fragmented and RNA samples were subjected to cDNA synthesis, followed by end-repair and A-tailing. Next, unique molecular identifiers were ligated and samples were barcoded. Two rounds of target capture, to allow maximal target enrichment, were followed by PCR amplification and sample purification. Finally, libraries were normalized and sequenced on a NextSeq 500 system (Illumina), combining 10 DNA libraries or 16 RNA libraries on a high-output or mid-output cassette, respectively. The raw sequencing data were processed andanalyzed by the TruSight Oncology 500 Local App version 1.3 and 2.0 (Illumina). Variants were filtered by the exclusion of (1) variants outside exons and splice site regions (−8/+8) except those in the *TERT* promoter region, (2) synonymous variants, unless present in a splice site region, (3) variants present with a frequency of > 0.1% in the ExAC (version 0.2) database, (4) variants with a variant allele frequency (VAF) of <5%, and (5) variants with <5 variant reads. Next, the remaining variants and gene amplifications in a virtual panel of 54 ‘actionable’ genes were further analyzed. This panel was established based on genes listed as biomarker for solid tumors in OncoKB, most recent literature and expert opinion of in-house consulted medical oncologists (Supplementary Data 4). Variants were manually inspected, curated, and classified based on predicted pathogenicity into 5 classes as described previously^21^: class 1, not pathogenic; class 2, unlikely pathogenic; class 3, variant of unknown significance; class 4, likely pathogenic; and class 5, pathogenic. Variants classified as 4 and 5 were considered as potentially clinically relevant. Therapeutic targeting of tumor suppressor genes (TSG) typically requires the inactivation of both gene copies. Therefore, for TSG we evaluated whether class 4 and 5 aberrations affect one or two alleles based on relative coverage and/or VAF of the variant and nearby single-nucleotide polymorphisms (SNPs). Analysis of TMB was based on both synonymous and nonsynonymous variants.

### Histopathological review, tissue microarray, and immunohistochemistry

Cases were histopathologically reviewed by a pathology team (R.S.v.d.P. and M.E.V.B) according to the WHO histologic classification of tumors of the gallbladder (5th ed.)^35^ and the American Joint Committee on Cancer tumor-node-metastasis classification system (8th ed.)^36^.

For all cases for whom tissue blocks were available, three representative areas were selected using hematoxylin and eosin (H&E) slides to construct tissue microarrays. Tissue microarrays (TMA’s) were constructed using a Quick-Ray manual tissue microarrayer (Quick-Ray, Unitma, Seongnam‐si, Korea). The three areas of each case were divided over three replicate TMA’s (A, B, C), each comprising 58 individual tumor cores and 2 reference cores of 2.0 mm. IHC was performed initially only on one tumor core of each case (TMA A).

IHC was performed essentially as described previously^37^ with standard chromogenic horseradish peroxidase-diaminobenzidine (HRP-DAB) detection method using the semiautomated LabVision Autostainer (Immunologic, Duiven, the Netherlands). IHC was performed both to aid in histological subtyping of GBC (EMA, MUC2, MUC5AC, MUC6, CK7, CK20, and p63), to validate NGS results and to screen for a selection of potential therapeutic targets in a large cohort of patients (PD-L1, HER2, Pan-TRK and p53). Details of antibodies are specified in Supplementary Data 5.

Expression of EMA, MUC2, MUC5AC, MUC6, CK7, and CK20 was classified into three categories: negative (<1% positive staining), intermediate (1–49% positive staining) or positive (≥50% positive staining). Expression of p63 was scored as negative or positive, and expression of p53 was scored as wildtype (only a few positive cells or with variable intensity) or aberrant (either complete loss of expression or overexpression). PD-L1 expression was assessed by the tumor proportion score (TPS) and categorized into four categories: TPS < 1%, TPS 1–10%, TPS 11–50% and TPS > 50%. HER2 expression was classified into four categories: negative (score 0), low (score 1+), intermediate (score 2+), and high (score 3+). An HER2 IHC score of 2+ was considered equivocal and a score of 3+ was considered as overexpression. Pan-TRK expression was scored as negative (<1% staining) or positive (≥1% staining); in the case of positive staining, the result was validated by the Archer FusionPlex lung panel assay in our diagnostic laboratory (ArcherDX, Boulder, CO, USA).

### Statistical analyses

Patient and tumor characteristics were described using counts and percentages. Differences between histological and molecular subgroups were assessed by the Χ^2^-test. For survival analyses, overall survival (OS) was defined as the interval in months between GBC diagnosis and time of death or last follow-up (01-02-2017). Patients alive at the last date of follow-up were censored. Survival curves were made according to the Kaplan–Meier method with log-rank testing to compare survival distributions.

All tests of significance were two-tailed and *P*-values of <0.05 were considered statistically significant. Statistical analyses were performed using IBM SPSS Statistics (version 22.0.0.1, IBM, Armonk, NY, USA).

### Reporting summary

Further information on research design is available in the Nature Research Reporting Summary linked to this article.

## Supplementary information

REPORTING SUMMARY

Supplemental Data

## Data Availability

Binary Alignment Map (BAM) and corresponding (annotated) variant call format (VCF) files of patients cannot be publicly shared under the obtained institutional review board approval, as patients were not consented to share raw sequencing data beyond the research and clinical terms. However, all variants assessed as (potentially) clinically relevant are presented in this paper and its supporting files. The datasets generated and analyzed during the current study are available from the corresponding author on reasonable request and upon a data usage agreement.
