# Supplementary material for: Comprehensive clinicopathological and genomic profiling of gallbladder cancer reveals actionable targets in half of patients
Source: NPJ Precis Oncol. 2022 Nov 5;6:83. doi: 10.1038/s41698-022-00327-y (PMC9637208; doi:10.1038/s41698-022-00327-y)
Supplement: Supplementary file 1 — REPORTING SUMMARY [file 41698_2022_327_MOESM1_ESM.pdf]

# nature portfolio

Corresponding author(s): Tessa J.J. de Bitter

Last updated by author(s): Oct 3, 2022

# Reporting Summary

Nature Portfolio wishes to improve the reproducibility of the work that we publish. This form provides structure for consistency and transparency in reporting. For further information on Nature Portfolio policies, see our Editorial Policies and the Editorial Policy Checklist.

## Statistics

For all statistical analyses, confirm that the following items are present in the figure legend, table legend, main text, or Methods section.

| n/a | Confirmed | |
|---|---|---|
| ☐ | ☒ | The exact sample size (*n*) for each experimental group/condition, given as a discrete number and unit of measurement |
| ☒ | ☐ | A statement on whether measurements were taken from distinct samples or whether the same sample was measured repeatedly |
| ☐ | ☒ | The statistical test(s) used AND whether they are one- or two-sided<br>*Only common tests should be described solely by name; describe more complex techniques in the Methods section.* |
| ☒ | ☐ | A description of all covariates tested |
| ☒ | ☐ | A description of any assumptions or corrections, such as tests of normality and adjustment for multiple comparisons |
| ☐ | ☒ | A full description of the statistical parameters including central tendency (e.g. means) or other basic estimates (e.g. regression coefficient) AND variation (e.g. standard deviation) or associated estimates of uncertainty (e.g. confidence intervals) |
| ☒ | ☐ | For null hypothesis testing, the test statistic (e.g. *F*, *t*, *r*) with confidence intervals, effect sizes, degrees of freedom and *P* value noted<br>*Give P values as exact values whenever suitable.* |
| ☒ | ☐ | For Bayesian analysis, information on the choice of priors and Markov chain Monte Carlo settings |
| ☒ | ☐ | For hierarchical and complex designs, identification of the appropriate level for tests and full reporting of outcomes |
| ☒ | ☐ | Estimates of effect sizes (e.g. Cohen's *d*, Pearson's *r*), indicating how they were calculated |

*Our web collection on statistics for biologists contains articles on many of the points above.*

## Software and code

Policy information about availability of computer code

| | |
|---|---|
| Data collection | No software was used for data collection. The raw sequencing data were processed and analyzed by the TruSight Oncology 500 Local App version 1.3 and 2.0 (Illumina). |
| Data analysis | Statistical analyses were performed using IBM SPSS Statistics (version 22.0.0.1, IBM, Armonk, NY, USA). |

For manuscripts utilizing custom algorithms or software that are central to the research but not yet described in published literature, software must be made available to editors and reviewers. We strongly encourage code deposition in a community repository (e.g. GitHub). See the Nature Portfolio guidelines for submitting code & software for further information.

## Data

Policy information about availability of data

All manuscripts must include a data availability statement. This statement should provide the following information, where applicable:

- Accession codes, unique identifiers, or web links for publicly available datasets
- A description of any restrictions on data availability
- For clinical datasets or third party data, please ensure that the statement adheres to our policy

Binary Alignment Map (BAM) and corresponding (annotated) variant call format (VCF) files of patients cannot be shared under the obtained institutional review board approval, as patients were not consented to share raw sequencing data beyond the research and clinical terms. However, all variants assessed as (potentially) clinically relevant are presented in this paper and its supporting files.

## Human research participants

Policy information about studies involving human research participants and Sex and Gender in Research.

| | |
|---|---|
| Reporting on sex and gender | Both tumor material from male and female individuals was used in this study, as gallbladder cancer (GBC) occurs in both sexes. Baseline information on sex was provided by the Netherlands Cancer Registration and supplemented with information from the original (anonymized) pathology reports. More female patients were included in the study than male patients since gallbladder cancer shows a female predominance. We have no reason to assume that findings apply to only one sex. |
| Population characteristics | All relevant characteristics are described in Table 1 of the manuscript. Median age was 69 years, 73% of the patients was female and all patients were diagnosed with pathology confirmed primary GBC |
| Recruitment | Patients that had undergone a resection for GBC between 2000 and 2019 were anonymously selected using the linkage between the nationwide network and registry of histopathology and cytopathology in the Netherlands (PALGA, LZV2017-87) and the Netherlands Cancer Registry (NCR, K171236). |
| Ethics oversight | This study was approved by the local Radboud university medical center medical ethics committee (2018-1426). |

Note that full information on the approval of the study protocol must also be provided in the manuscript.

# Field-specific reporting

Please select the one below that is the best fit for your research. If you are not sure, read the appropriate sections before making your selection.

☒ Life sciences ☐ Behavioural & social sciences ☐ Ecological, evolutionary & environmental sciences

For a reference copy of the document with all sections, see nature.com/documents/nr-reporting-summary-flat.pdf

# Life sciences study design

All studies must disclose on these points even when the disclosure is negative.

| | |
|---|---|
| Sample size | No sample size was calculated. As GBC is a very rare cancer, we collected anonymized patient information and resection specimens from a nationwide cohort of patients using our two nationwide databases: PALGA (LZV2017-87) and the Netherlands Cancer Registry (NCR, K171236). Our cohort is novel in terms of size and we believe that the sample size is justified for the conclusions that were drawn. |
| Data exclusions | Figure 1 describes the flow chart of the study. Only patients for which the pathology report, original H&E slides and/or FFPE tissue blocks were available were included. Next, patients for which resection specimens were of insufficient quality or quantity for subsequent analyses were excluded. Additionally, patients with a neuroendocrine GBC (N=10), a very rare subgroup of GBC, were described elsewhere and were therefore excluded from this study. |
| Replication | Sequencing was only performed once. For a subset of targets, sequencing data was validated on the protein level by immunohistochemistry and results correlated well. |
| Randomization | This is not relevant to the study. We only aimed to give a descriptive overview of actionable molecular targets in GBC. |
| Blinding | This is not relevant to the study. We only aimed to give a descriptive overview of actionable molecular targets in GBC. |

# Reporting for specific materials, systems and methods

We require information from authors about some types of materials, experimental systems and methods used in many studies. Here, indicate whether each material, system or method listed is relevant to your study. If you are not sure if a list item applies to your research, read the appropriate section before selecting a response.

## Materials & experimental systems

| n/a | Involved in the study | |
|---|---|---|
| ☐ | ☒ | Antibodies |
| ☒ | ☐ | Eukaryotic cell lines |
| ☒ | ☐ | Palaeontology and archaeology |
| ☒ | ☐ | Animals and other organisms |
| ☐ | ☒ | Clinical data |
| ☒ | ☐ | Dual use research of concern |

## Methods

| n/a | Involved in the study | |
|---|---|---|
| ☒ | ☐ | ChIP-seq |
| ☒ | ☐ | Flow cytometry |
| ☒ | ☐ | MRI-based neuroimaging |

## Antibodies

Antibodies used

All antibodies that were used are extensively validated and routinely used in our diagnostics laboratory.

PD-L1:
- Citation: Sacher AG, et al. JAMA Oncol. 2016; 2:1217-22.
- Supplier: Agilent Dako
- Cat no.: M365329-2
- Clone no.: 22C3

pan-TRK:
- Citation: Xu B, et al. Histopathology. 2020; 76:375-382
- Supplier: Abcam
- Cat no: Ab181560
- Clone no: EPR17341

HER2:
- Citation: Graziano C. CAP Today. 1998; 12:13-16.
- Supplier: Agilent Dako
- Cat no: SK00121-2
- Clone no: ready to use

p53:
- Citation: Moore BE, et al. Appl Immunohistochem Mol Morphol. 2001; 9:203 –206.
- Supplier: Immunologic (VWR international)
- Cat no: ILM27011-C1
- Clone no: D07

EMA:
- Citation: Langner C, et al. Mod Pathol. 2004; 17:180-8
- Supplier: Agilent Dako
- Cat no: M061301-2
- Clone no: E29

MUC2:
- Citation: Mino-Kenudson M, et al. Arch Pathol Lab Med. 2007; 131:86-90.
- Supplier: Cell Marque (Sanbio)
- Cat no: 291M-16
- Clone no: MRQ-18

MUC5AC:
- Citation: Lau SK, et al. Am J Clin Pathol. 2004; 122:61-9.
- Supplier: Cell Marque (Sanbio)
- Cat no: 292M-96
- Clone no: MRQ-19

MUC6:
- Citation: Mino-Kenudson M, et al. Virchows Arch. 2016; 469:255-65.
- Supplier: Cell Marque (Sanbio)
- Cat no: 293M-95
- Clone no: MRQ-20

CK7:
- Citation: Jerome MV, et al. Histopathology. 2004; 45:125-34.
- Supplier: Cell Marque (Sanbio)
- Cat no: 307M-95
- Clone no: OV-TL 12/30

CK20:
- Citation: Van der Linden M et al. Hum Pathol. 2017; 68:184-188.
- Supplier: Immunologic (VWR international)
- Cat no: ILM2133-C1
- Clone no: E19-1

p63:
- Citation: Werling RW, et al. Am J Surg Pathol. 2003; 27:82-90.
- Supplier: Immunologic (VWR international)
- Cat no: ILM8651-C1
- Clone no: 4A4

Validation

All antibodies that were used are extensively validated and routinely used in our diagnostics laboratory. Moreover, the antibodies are all are described in literature, of which an example reference is cited above.

# Clinical data

Policy information about clinical studies

All manuscripts should comply with the ICMJE guidelines for publication of clinical research and a completed CONSORT checklist must be included with all submissions.

| | |
|---|---|
| Clinical trial registration | We only include retrospective, anonymized clinicopathological data provided by two nation-wide databases: PALGA (LZV2017-87) and the Netherlands Cancer Registry (NCR, K171236) |
| Study protocol | We only include retrospective, anonymized clinicopathological data provided by two nation-wide databases: PALGA (LZV2017-87) and the Netherlands Cancer Registry (NCR, K171236) |
| Data collection | We only include retrospective, anonymized clinicopathological data provided by two nation-wide databases: PALGA (LZV2017-87) and the Netherlands Cancer Registry (NCR, K171236) |
| Outcomes | We only include retrospective, anonymized clinicopathological data provided by two nation-wide databases: PALGA (LZV2017-87) and the Netherlands Cancer Registry (NCR, K171236). For survival analyses, overall survival (OS) was defined as the interval in months between GBC diagnosis and time of death or last follow-up (01-02-2017) |
